# Role of p16 deletion and Bmi1 copy number variation in glioma

**DOI:** 10.1186/1755-8166-7-S1-P11

**Published:** 2014-01-21

**Authors:** GK Chetan, M K Sibin, Dhananjaya I Bhat, Ch Lavanya, Manoj M Jeru, N Geethashree

**Affiliations:** 1 Department of Human genetic, National Institute of Mental Health and neurosciences, Hosur road, Bangalore-29, India; 2 Department of Neurosurgery, National Institute of Mental Health and neurosciences, Hosur road, Bangalore-29, India

## Background

Malignant gliomas are the most common and lethal intracranial tumors. Glioma exhibit a relentless malignant progression characterized by widespread invasion throughout the brain, resistance to traditional and newer targeted therapeutic approaches. The classical genetic alterations in glioma target pathways governing cellular proliferation, cellular survival (apoptosis and necrosis), invasion, and angiogenesis. The p16 is the second most altered tumor suppressor gene and frequent mutations and deletions of p16 in human cancer cell lines first suggested an important role for p16 in carcinogenesis. The Bmi-1 is an important oncogene and its expression is found to be elevated in many types of cancers. Role of Bmi1 copy number variation in glioma is still under debate. In this study we analyzed the alterations in p16 and Bmi1 genes in glioma.

## Material and methods

50 glioma samples from patients were collected from the neurosurgery OT. Tissues having >95% tumor cells were processed and DNA was isolated. For the analysis of p16 deletion multiplex PCR was done using primers specific for all 3 exons of p16. Copy number variation in Bmi1 gene was analyzed using real time PCR with Bmi1 specific primers.

## Results

Our results showed that there is 20% of p16 deletion in our samples and the deletion pattern vary with exons. There was no copy number variation found in Bmi1 gene in glioma.

## Conclusions

We concluded that the p16 deletion is a common alteration found in glioma and Bmi1 gene amplification is not the common mechanism to increase the expression of this protein in glioma.

